# Assessing Change in Sleep Latency as a Potential Indicator of Alzheimer’s Pathologies in Essential Tremor

**DOI:** 10.1093/geroni/igaf122.3174

**Published:** 2025-12-31

**Authors:** Hasib Mia, Silvia Chapman, Sandra Rizer, Phyllis Faust, Elan Louis, Stephanie Cosentino

**Affiliations:** Columbia Presbyterian Medical Center, New York, New York, United States; Columbia University Irving Medical Center, New York, New York, United States; Columbia University Irving Medical Center, New York, New York, United States; New York-Presbyterian Hospital, New York, New York, United States; UT Southwestern Medical Center, Dallas, Texas, United States; Columbia University Irving Medical Center, New York, New York, United States

## Abstract

Sleep disturbances, including prolonged sleep latency, are frequently reported in Essential tremor (ET) and other neurodegenerative conditions, yet their underlying etiology remains unclear. This study examines whether an increase in sleep latency over time—measured by the Pittsburgh Sleep Quality Index (PSQI)—is associated with tau pathology and amyloid burden in older adults with ET. In a sample of 24 older adults, we assessed changes in sleep latency over time in relation to Alzheimer’s disease (AD) neuropathological outcomes, including amyloid plaques and neurofibrillary tangles (tau). Tau pathology was estimated using Braak Stage (AD), and amyloid burden through CERAD scores. Pearson correlations and multiple regression analyses were conducted, adjusting for age, education, and time between sleep data collection and death. Correlational findings indicate an association between increased sleep latency and tau pathology (r = 0.473, p = 0.023) and amyloid burden (r = 0.448, p = 0.028). However, when examined in an adjusted regression, the change in sleep latency was not associated with pathological outcomes (tau: β = 0.129, p = 0.664; amyloid: β = 0.257, p = 0.324). Results suggest that while sleep latency may be linked to neurodegenerative pathology, additional factors contribute to this relationship, warranting further research. This study contributes to the growing body of literature on sleep disturbances in dementia by highlighting the influence of confounding factors. Understanding these relationships may help establish sleep latency as a diagnostic tool or biomarker for neurodegenerative conditions, while emphasizing the need for a more nuanced interpretation of its predictive value.

